# Effect of Supplemental Language Therapy on Cortical Neuroplasticity and Language Outcomes in Children with Hearing Loss

**DOI:** 10.3390/brainsci15020119

**Published:** 2025-01-26

**Authors:** Anu Sharma, Kayla Cormier, Jim Grigsby

**Affiliations:** 1 Department of Speech Language and Hearing Sciences, University of Colorado Boulder, 2501 Kittredge Loop Dr. UCB 409, Boulder, CO 80309, USA; kayla.cormier@colorado.edu; 2 Department of Psychology, University of Colorado Denver, Campus Box 173, P.O. Box 173364, Denver, CO 80217, USA; jim.grigsby@ucdenver.edu

**Keywords:** auditory evoked potentials, language therapy, cochlear implant, hearing aids, pediatric, hearing loss

## Abstract

Background/Objectives: The cortical auditory evoked potential P1 response is a biomarker of cortical auditory maturation for tracking longitudinal cortical maturation in children with hearing loss after treatment with hearing aids and/or cochlear implants. In conjunction with hearing treatments, children with hearing loss commonly receive language therapy services. However, the effect of language therapy on cortical maturation in hearing loss is less well studied. Methods: This study explored auditory cortical maturation changes, using the P1 response, with coinciding language changes, utilizing the Preschool Language Scales test, following approximately six months of supplemental listening and spoken language therapy services in 39 children with hearing aids or cochlear implants. Results: Following supplemental language therapy, P1 latencies significantly decreased in all children, at a rate found to be significantly faster than expected for typical maturation. Language scores also significantly improved beyond expected maturation effects and were correlated with P1 latencies following supplemental therapy. Overall, with approximately six months of therapy, the children in this study made significantly greater gains of 9 to 10 months in total language and expressive communication. A subgroup analysis revealed that children with cochlear implants showed significantly lower language scores than their chronological age following supplemental therapy, while children with hearing aids obtained language scores that were not significantly different to their chronological age at follow-up. Conclusions: Overall, the results from this study showed that supplemental language therapy resulted in more typical auditory cortical maturation and improved language abilities and that the P1 CAEP response can objectively track neuroplastic changes in children as a function of language therapy provided in conjunction with hearing aids and CIs.

## 1. Introduction

In the United States, the prevalence rate of childhood hearing loss is 0.6% [1]. Children with mild-moderate permanent sensorineural hearing loss are typically treated with hearing aids and those with severe-profound hearing losses are typically fit with cochlear implants (CIs). While hearing aids amplify sounds based on an individual’s hearing loss, CIs function by electrically stimulating the auditory nerve directly through an electrode array placed within the inner ear. Given that young children cannot have their hearing tested using conventional techniques which require a behavioral response, objective measures of hearing loss are essential for monitoring hearing treatments in children. Research has shown that the cortical auditory evoked potential (CAEP) P1 response is an objective EEG biomarker of auditory cortical maturation, reflecting maturation of the primary auditory cortex. The P1 response is an obligatory response present at birth with extrinsically driven (e.g., through auditory input) developmental neuroplastic changes that have been well documented in normal hearing children [2,3,4]. Through this research, Sharma and colleagues have established normative values with 95% confidence intervals of P1 latencies with respect to age in children without hearing loss [2,3,4]. Therefore, P1 responses from children with hearing loss can be compared to these age based normative values to determine if a child’s auditory cortical maturation is appropriate for their age or delayed for their age [2,3,4,5,6,7,8,9]. This comparison of P1 latencies to age-matched normative values has been primarily studied as a function of hearing treatment use including hearing aids and CIs [5,6,8,10,11,12,13,14]. Comparing children with and without hearing loss is particularly advantageous, given a goal of hearing intervention is to close the gap between children with and without hearing loss on auditory and language measures. Early intervention in children with hearing loss has been shown to result in a higher portion of CI children scoring within normative ranges on language abilities [15,16,17].

The use of P1 responses in guiding hearing loss treatment decisions was exemplified in a case study by Sharma et al. [10]. In this case study, a child fit with bilateral hearing aids at 21 months of age obtained CAEP testing due to significant delays in speech and language development. With hearing aids, the child showed a delayed P1 response for their age. Combining these results with other clinical outcomes, the family decided to pursue CIs, and the child was implanted at 25 months of age. Following several months of CI usage, the child returned for CAEP testing and P1 latencies fell within age-matched normative limits co-incident with improvement in speech and language development [10]. Thus, P1 CAEP responses can be a useful tool for objectively assessing hearing treatment.

In addition to hearing treatment with devices, CAEP responses have been examined in CI children undergoing a computerized auditory training program. Following the training program the children showed reductions in P1 latencies, however, children who did not receive the intervention also demonstrated reductions in P1 latencies over this time, likely due to age-related maturation [18]. Given that P1 latencies are expected to decrease with maturation, studies that take into account age-related (or normative) developmental effects in addition to auditory/language therapies on CAEP responses are warranted.

Children with hearing loss typically receive speech-language therapy in addition to using hearing devices which may affect CAEP responses, as previous research has demonstrated CAEP measures are correlated with language skills [7,8,9,19]. On average longer P1 latencies have been related to poorer language abilities, while shorter P1 latencies, closer to that of age-appropriate responses, have been associated with better language outcomes [7,8,9,19]. Language therapy, a primary target of early intervention, leads to better language outcomes for children with hearing loss [20]. However, in a recent study of children with treated hearing loss, it was noted that, on average, even in children with treated hearing loss over half were below normative standards on expressive and receptive language skills [21]. In comparing children with hearing loss who obtained speech-language therapy to children with hearing loss who did not, children who had undergone an 11-week speech-language and communication intervention showed better standardized language scores [22]. Furthermore, research by Jackson and Schatschneider [23] has shown that auditory–verbal language therapy (AVT) can result in significantly increased language scores with the amount of therapy being related to increases in language scores. However, even with AVT, children continued to fall outside of normative language values for their age in that study [23].

Additionally, factors such as the degree of hearing loss and amount of audibility provided by hearing treatments have been shown to have a large impact on both cortical maturation and language development [24]. Prior research into language development has examined differences in language abilities between children with hearing aids versus CIs with mixed results on the impact of hearing devices on language. When examining the same degree of hearing loss, no significant differences were noted between hearing aid users and CI users on receptive and expressive language skills [25,26,27]. Additionally, speech intelligibility has been noted to not significantly differ between hearing treatment devices [28]. However, one study found children with bilateral hearing aids were more likely to have worse receptive vocabulary and phonological skills than bilateral CI users [21]. Using parental report as an outcome, CI users have also been noted to show significantly better auditory perception than hearing aid users [28]. Furthermore, even within children who were implanted prior to the age of 3.5 years (considered to be within the sensitive period for auditory cortical maturation) there is variability in CI outcomes [29,30,31]. Therefore, it is useful to not only investigate the effects of language therapy on cortical maturation and language development, but to further examine these effects by hearing treatment devices.

The present study aimed to examine the effects of supplemental language therapy in children with hearing aids and CIs, whose language abilities on average were below age-norms, on auditory cortical maturation and language development. As there can be variability in hearing treatment outcomes, we wanted to focus specifically on children who were self-identified as having difficulties with language outcomes despite receiving hearing loss treatment at a young age. We hypothesized that supplemental language therapy would result in more appropriate auditory cortical maturation and improved language development. Additionally, the effects of supplemental language therapy intervention were examined by hearing treatment device (hearing aids vs. CIs).

## 2. Materials and Methods

This study employed a pretest-posttest design. The design of this study was retrospective, as data was collected as part of a larger study approved by the Colorado Multiple Institutional Review Board (COMIRB).

### 2.1. Participants

A total of 41 English-speaking children, aged between 20 months and 78 months, were included in the study. All children were English-speaking as the measures used in this study were administered in English. All participants exhibited bilateral moderate to profound sensorineural hearing loss, treated with either behind-the-ear (BTE) hearing aids or CIs. Children were defined as CI users if they utilized one CI with or without a hearing aid in the other ear or had bilateral CIs. Thus, the children were categorized into two groups based on hearing loss treatments, with 20 children utilizing hearing aids and 21 children utilizing CIs. Two children were removed from analysis. One of these children transitioned from utilizing bilateral hearing aids to CIs during the study, and another child with CIs presented with an artifact from the CIs, which obscured their P1 test results. This resulted in 19 children with hearing aids and 20 children with CIs being included in the analysis. All children completed language testing pre- and post-intervention. Two children completed P1 testing only post-intervention.

Independent sample *t*-tests were utilized to verify that when split into treatment device type groups the children did not significantly differ in terms of age or hearing rehabilitation characteristics. Aided testing revealed the hearing aid group demonstrated significantly worse aided hearing abilities than the CI group (t(24.08) = 2.92, *p* = 0.008). Independent sample *t*-tests and two proportion z-tests were performed to ensure that there were no significant differences between the groups in terms of demographics. There were no significant differences in age, gender, or maternal education. Moreover, there were no significant differences in parental reports of disabilities in addition to the hearing loss with 36.84% of hearing aids users and 55% of CI users having an additional disability. Finally, the majority of children in this study self-reported at least some use of sign language (81%), with there being no significant differences in the percentages of hearing aid users and CI users who used some sign language. None of the children enrolled in this study reported that the main language used in the home was sign language. Participant demographic information can be viewed in Table 1.

### 2.2. Intervention

All the children included in this study received supplemental listening and spoken language therapy (LSL) services in addition to any speech language services received via early intervention or school departments. LSL therapy emphasizes the use of hearing devices to develop oral expressive and receptive language skills in children with hearing loss. LSL therapy provides a family centered approach and teaches parents skills to promote speech and language development within the home. LSL therapy is a widely used language therapy and has been shown to have a positive effect on language development in children with hearing loss [32,33,34,35,36]. The LSL therapy intervention in this study was provided either in person or via telehealth. Recent research by Grigsby and colleagues [37] has shown that there are no significant differences in language outcomes from LSL therapy provided via telehealth or in-person, suggesting telehealth was noninferior to in-person LSL therapy [37]. Language therapy services were provided by experienced teachers of the deaf. Months of therapy ranged from 4.37 to 6.67 months with the average months of therapy being 5.79 months. Independent sample *t*-tests and two proportion z-tests confirmed no significant differences were present between the device groups in the language therapy delivery method or in the length of therapy (see Table 1).

### 2.3. Measures

#### 2.3.1. P1 Cortical Auditory Evoked Potential

The P1 Cortical Auditory Evoked Potential (CAEP) biomarker was recorded to evaluate cortical auditory development, with data collected pre-supplemental language therapy and post-supplemental language therapy which occurred on average within half a day (SD = 48 days) and two days (SD = 11 days) of baseline and follow-up testing for language assessments, respectfully. CAEPs were recorded in response to the synthesized speech syllable /ba/ with a stimulus duration of 90 ms. This well-established stimulus has been consistently used in previous studies [17,18,30]. The stimulus was presented at 65 dB HL through two loudspeakers positioned at 45° azimuth.

Testing took place in an electromagnetically shielded sound booth, with the child seated on a parent’s lap in a comfortable chair. Children watched a silent movie of their choice on a TV monitor in front of them. The entire test session, encompassing electrode application and CAEP recordings, lasted approximately an hour. Throughout the testing, the subjects’ hearing aids or cochlear implants were set to each child’s regular everyday settings.

Brain responses were measured using 5–8 electrodes, depending on whether the child had a hearing aid or cochlear implant. To minimize CI artifact, eight electrodes along the isopotential contours were used for children with CIs, following a previously published method [38]. The active electrode was positioned at Cz, with a ground electrode at Fpz. NeuroScan acquisition software (Compumedics Neuroscan) was employed for recording and analyzing cortical responses. Raw data was sampled at 1000 Hz and filtered from 0.1 to 100 Hz. Epochs contaminated by eye movements or external artifacts (over ±100 µV) were rejected.

Two children, one with a CI and one with hearing aids, did not complete baseline testing. To ensure response replicability, at least two runs were conducted, and a grand average of two replicable responses was generated to determine P1 peak latencies. Participants who did not complete both testing sessions were excluded from analyses examining the change in P1 latencies over the course of the study. P1 responses were compared to age-matched normative values and were categorized as abnormal if the response was not replicable or if the latency was outside the upper 95% confidence interval of the normative latency.

#### 2.3.2. Language Outcomes

An assessment of language skills was conducted using the Preschool Language Scales 5th Edition (PLS-5). The PLS-5 is a play-based test battery of language development, designed for children up to seven years of age. This measure included scores for Auditory Comprehension (AC), Expressive Communication (EC), and a Total Language Score. Raw scores were converted to age-equivalent scores in months, as outlined by Zimmerman et al. [39] and Sahli & Belgin [40]. In addition to age-equivalent scores, growth scale value scores were obtained for AC and EC and standardized scores were calculated for AC, EC, and Total Language based on raw score conversions [39]. The baseline appointment occurred at the time of enrollment prior to the beginning of the intervention, and follow-up appointments occurred following the end of supplemental language therapy. On average, follow-up testing occurred 6.74 months (SD = 22 days) after baseline testing. Baseline assessment of language on average occurred 25 days (SD = 59 days) prior to the start of supplemental language therapy, while follow-up testing on average took place 13 days (SD = 13 day) after the end of supplemental therapy.

#### 2.3.3. Aided Hearing Outcomes

Aided testing with hearing aids and CIs was completed for each child at their P1 CAEP follow-up visit. Given that the children included in this study ranged in age, a variety of conventional aided testing techniques were utilized. Aided testing included children responding to beeps at different frequencies, repeating back speech, or responding to speech at various intensity levels. Thresholds were determined as the lowest sound intensity level in which the child could respond 50% of the time. For children that responded to beeps, the responses from 500, 1000, and 2000 Hz were averaged together to create a three-frequency pure tone average (PTA). Some children completed aided testing in each ear separately while others completed tasks binaurally. For children that were able to complete aided testing in each ear separately, the responses from each ear were averaged to create one aided value for each child. All aided testing was completed utilizing hearing treatments using the children’s daily settings.

### 2.4. Statistical Analysis

The statistical analysis was performed using R (version 4.2.1) and RStudio. Non-parametric Fisher’s Exact Tests were employed to assess the distribution of children with abnormal and normal P1 responses compared to age-matched normative values for each group. Age equivalent PLS-5 scores were compared to chronological age using one sample *t*-tests. Analyses were conducted for pre-language therapy measures, post-language therapy measures, and the change in outcomes pre- and post-intervention. Change in chronological age and change in upper, 95% confidence interval normative latencies were utilized in the comparison of the change in their respective measures using one sample *t*-tests. Paired *t*-tests were utilized to examine pre- to post-intervention changes in P1 latencies and language development. Growth scale value scores and standardized scores were used in these paired *t*-tests in addition to age-equivalent scores to fully examine how language skills changed from pre- to post-intervention. Analyses were conducted collapsing across all children, as well as sub-setting the data by hearing device. This allowed for further examination of how children with hearing aids and CIs may differ. In addition to the one-sample *t*-tests and paired *t*-test described above, two-tailed independent sample *t*-tests were used to assess group differences between children with hearing aids and children with CIs in the subgroup analyses. T-tests with unequal variances were used when appropriate, based on a test of the variance. Finally, spearman correlations were examined between all PLS-5 age equivalent scores and P1 latencies at baseline and follow-up for all children, regardless of hearing device.

Test residuals were used to identify outliers using Tukey’s method, where any subject with residuals above or below the third quartile was considered an outlier. Three subjects, two hearing aid users, and one CI user were identified as outliers in at least one test. One hearing aid subject was an outlier in four of the comparisons, one hearing aid subject was an outlier in 2 comparisons, and one CI subject was an outlier in 1 comparison.

Following removal of outliers, assumptions of normality were assessed by examining the model residuals via quantile–quantile plots. Violations of normality were found in the following comparisons:

1. All PLS-5 age equivalent scores versus chronological age pre-language therapy for hearing aid users.
2. PLS-5 growth scale value EC scores pre-language therapy versus post-language therapy for children with CIs.

For these comparisons, only non-parametric Wilcoxon Signed-Rank Tests were performed.

Post hoc power analyses with α equal to 0.05 (two-tailed) revealed power (1 − β) greater than 0.80 for paired sample tests and one sample *t*-tests when examining P1 latencies at each time point, changes in P1 latencies, change in all PLS-5 measures, and PLS-5 EC and total scores at baseline for all of the children as a group. One sample *t*-tests comparing chronological age to all PLS-5 measures post-intervention and PLS-5 AC pre-intervention were not found to be powered at 0.80 or greater. Therefore, both parametric and non-parametric Wilcoxon Signed-Rank Tests were performed for the post-intervention comparison of chronological age to PLS-5 age equivalent scores, as well as to PLS-5 AC age equivalent scores pre-intervention. Additionally, due to the small sample sizes for the subgroup analyses both parametric and non-parametric tests were completed. Non-parametric tests were utilized to limit the risk of type II errors. Parametric and non-parametric test results did not differ, except when comparing the PLS-5 AC age equivalent score to chronological age at follow-up. When parametric and non-parametric test results did not differ, only parametric tests results were reported given that they are more easily interpreted and commonly utilized in medical research.

## 3. Results

### 3.1. Changes in P1 Cortical Auditory Evoked Responses

**Figure 1:**
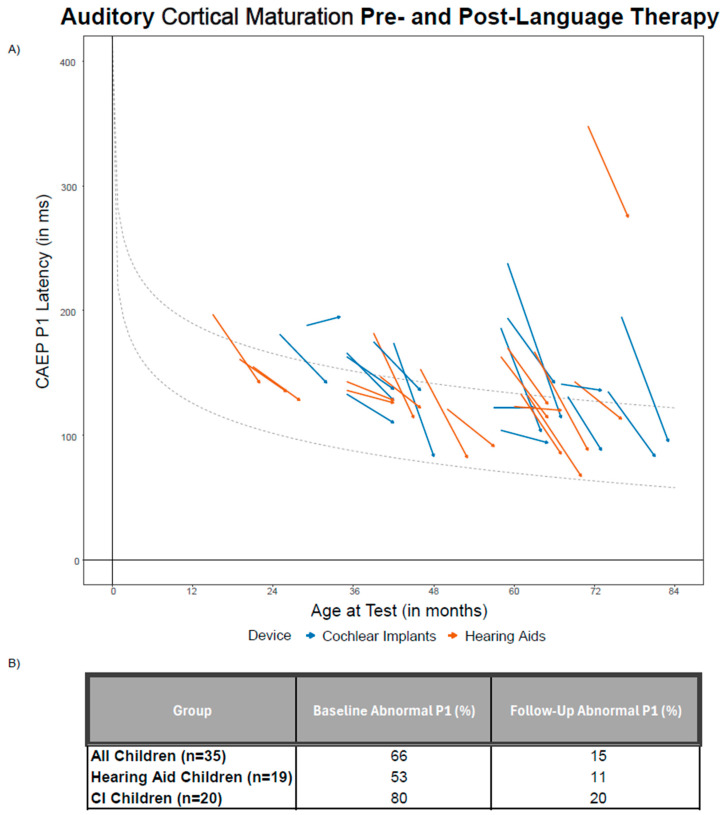
**Auditory Cortical Maturation Pre- and Post-Language Therapy.** (**A**). Confidence intervals of 95% for age-matched normative values are indicated by the light gray dashed lines. Straight lines show the improvement of individual latencies from pre-language therapy to post-language therapy: blue lines depict children with cochlear implants (CIs), and orange lines depict children with hearing aids. Overall, 48.72% of children in this study demonstrated a change from an abnormal to normal P1 response using age-matched normative values. (**B**). Table of percentage of children showing abnormal P1 responses prior to supplemental language therapy and following supplemental language therapy. There were no significant differences by hearing device in the percentage of children outside of age-matched normative values at baseline nor at follow-up.

As demonstrated in Figure 1A, overall the children showed a significant decrease in P1 latencies from pre- to post-intervention (t(32) = 8.11, *p* < 0.001). Four children, three CI users and one hearing aid user, demonstrated no replicable P1 responses at baseline. One child with bilateral CIs demonstrated a small 7 ms increase in P1 latency at follow-up, and another CI child demonstrated no change in P1 latency. When examining P1 responses categorically, the majority of the children had abnormal P1 responses at baseline (66.32%) consistent with their history of difficulties with speech-language development. However, Figure 1 demonstrates that following approximately six months of supplemental language therapy only 15.27% of the children continued to demonstrate abnormal P1 responses. Therefore, 48.72% of all children in this study exhibited a change from an abnormal P1 response at baseline to a normal P1 response for their age at follow-up. There were no significant differences in the distribution of children with abnormal or normal P1 responses for their age pre-language therapy, post-language therapy, or in the proportion of children exhibiting change from abnormal P1 responses to normal P1 response from pre- to post-language therapy between the hearing aid group and the CI group (see Figure 1B).

**Figure 2:**
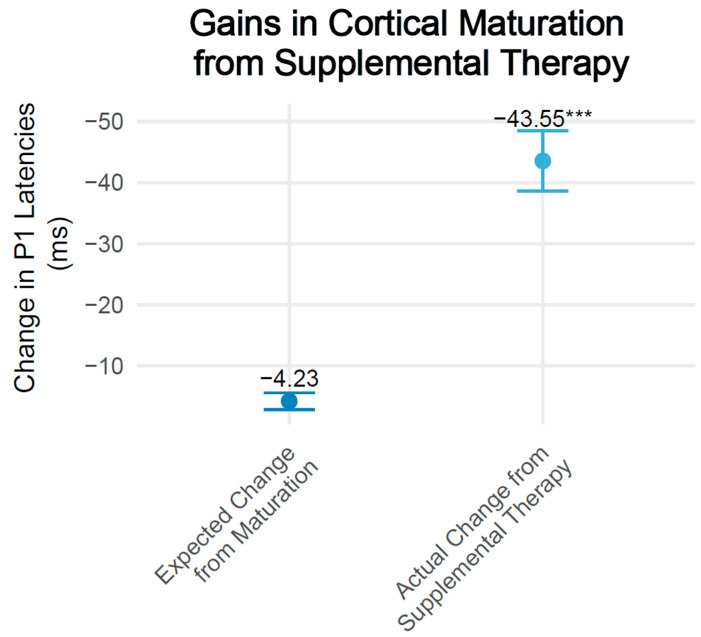
**Gains in cortical maturation from supplemental therapy.** The dark blue dot represents the mean change in P1 latencies expected due to maturation, while the light blue dot shows the mean change in P1 latencies following supplemental therapy. Error bars demonstrate the standard errors. Expected change in P1 latencies were determined using the 95% upper confidence levels of normative values. The average actual decrease in P1 latencies (left) was significantly greater than that expected from maturation (right) (*** *p* < 0.001).

As demonstrated in Figure 2, the change in P1 latencies for all of the children was greater than the expected change in P1 latencies from age-related maturation, as indexed by the change in upper, 95% confidence interval normative latencies for each child’s change in age over the duration of the study (t(32) = 7.10, *p* < 0.001). Sub-group analyses revealed both children with CIs (t(15) = 4.75, *p* < 0.001) and children with hearing aids (t(16) = 7.37, *p* < 0.001) showed a significant decrease in P1 latencies pre- to post-intervention, with no significant differences noted in the change in P1 latencies by device group. Children with CIs (M = −44.88 ms, SEM = ±9.45 ms; t(15) = 4.24, *p* = 0.001), and children with hearing aids (M = −42.29 ms, SEM = ±5.74 ms; t(16) = 6.31, *p* < 0.001) also exhibited changes in P1 latencies beyond those expected from maturation (CIs: M = −2.77 ms, SEM = ±2.59 ms; hearing aids: M = −5.78 ms, SEM = ±0.70 ms).

**Figure 3:**
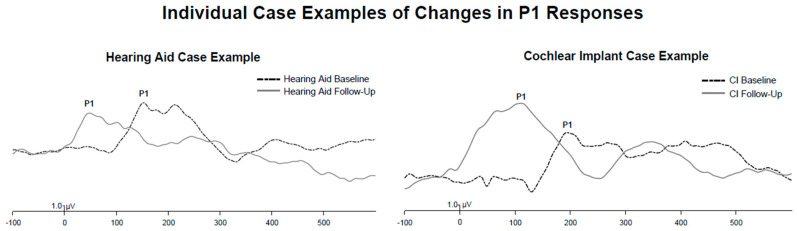
**Individual case examples of changes in P1 responses following supplemental language therapy.** Dashed lines correspond to baseline P1 CAEP responses, while solid lines indicate follow-up P1 CAEP responses. The change in P1 waveforms for a child with hearing aids is illustrated on the left, while the change in P1 waveforms for a child with CIs is demonstrated on the right. Both children exhibited a decrease in P1 latencies with supplemental language therapy.

At follow-up, only one child with a hearing aid was found to have no replicable P1 response. At follow-up, CI users and hearing aid users continued to show no significant differences between their P1 latencies. Figure 3 illustrates a case example of the change in P1 responses of a hearing aid and CI user.

### 3.2. Changes in Language Measures

**Figure 4:**
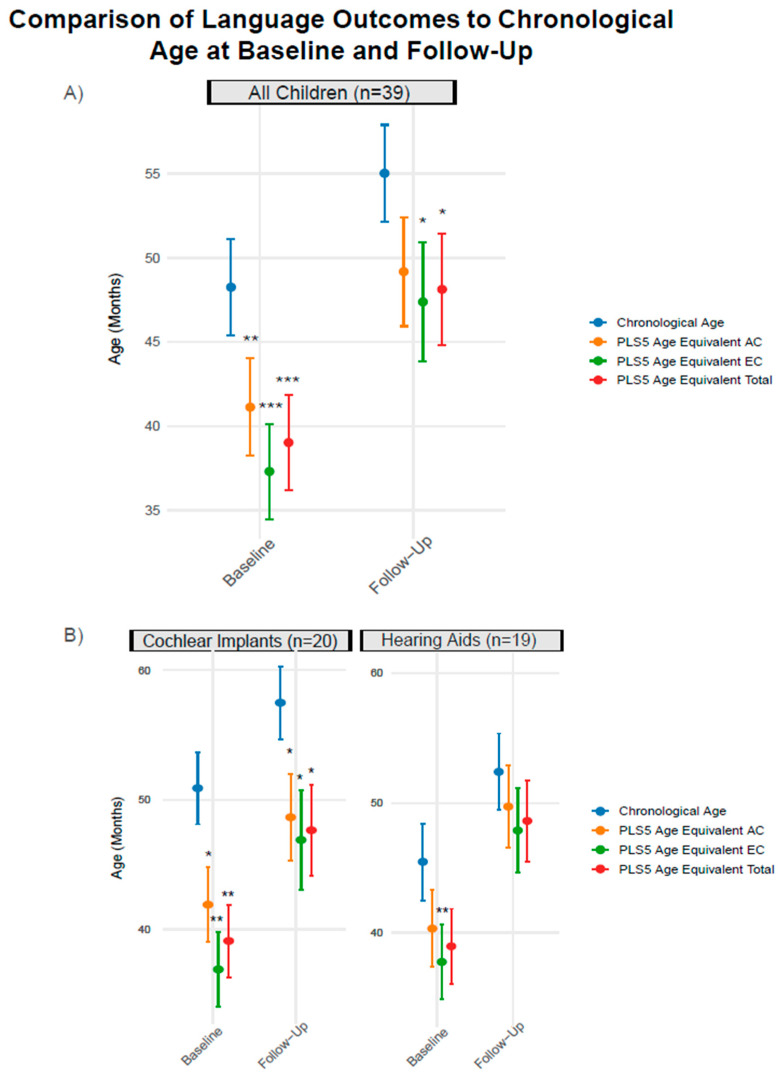
**Comparison of language outcomes to chronological age at baseline and follow-up.** Mean chronological ages and age equivalent PLS-5 language scores (AC, EC, and Total) are plotted as multicolored dots. The error bars extending from the dots indicate the standard errors of the mean. Differences between each PLS-5 age equivalent score with chronological age were tested prior to the children starting language therapy and following the completion of language therapy. (**A**). At baseline children in this study demonstrated significantly lower PLS-5 age equivalent scores compared to their chronological age. Following approximately six months of supplemental LSL therapy, the children continued to show significantly lower expressive communication (EC) and total language scores compared to their chronological age. (**B**). A subgroup analysis revealed that at baseline children with hearing aids showed only significantly lower age equivalent EC scores compared to their chronological age. Following supplemental therapy, the children with hearing aids no longer exhibited a significant difference between their language scores and chronological age. However, children with CIs demonstrated significantly lower age equivalent language scores compared to their chronological age at both pre- and post-intervention. *** *p* < 0.001, ** *p* < 0.01, * *p* < 0.05.

As shown in Figure 4A, prior to supplemental therapy the children demonstrated significantly lower age equivalent scores on PLS-5 AC scores (t(38) = 3.16, *p* = 0.003), PLS-5 EC scores (t(38) = 4.49, *p* < 0.001), and PLS-5 total language scores (t(38) = 4.10, *p* < 0.001) than their chronological age. The children demonstrated significant improvements in age equivalent PLS-5 AC scores (t(38) = 6.93, *p* < 0.001), PLS-5 EC scores (t(38) = 7.67, *p* < 0.001), and PLS-5 total scores (t(38) = 9.46, *p* < 0.001) following supplemental language therapy. Furthermore, a significant increase in PLS-5 growth scale values was observed for AC scores (t(38) = 6.28, *p* < 0.001) and EC scores (t(38) = 8.80, *p* < 0.001). Significant improvements in PLS-5 standardized scores were also found for EC scores (t(38) = 3.18, *p* = 0.003) and total scores (t(38) = 2.80, *p* = 0.008). No significant change in PLS-5 AC standardized scores was found. Although the children made significant language gains, following the supplemental language therapy age equivalent PLS-5 EC scores (t(38) = 2.64, *p* = 0.012) and total scores (t(38) = 2.54, *p* = 0.015) remained on average significantly lower than the children’s chronological age. Age equivalent PLS-5 AC scores did not significantly differ from the children’s chronological age post-supplemental language therapy (see Figure 4A).

In sub-group analyses by hearing device, CI children exhibited significantly lower PLS-5 age equivalent AC scores (t(19) = 2.68, *p* = 0.015), EC scores (t(19) = 3.93, *p* = 0.001), and total language scores (t(19) = 3.63, *p* = 0.002) than their chronological age before receiving the supplemental language therapy. As illustrated in Figure 4B, children with hearing aids showed only significantly lower PLS-5 age equivalent EC scores than their chronological age (z = −2.76, *p* = 0.006). However, there were no significant differences between children with CIs and hearing aids on pre-intervention PLS-5 age equivalent scores.

Children with CIs showed a significant change in PLS-5 age equivalent AC scores (t(19) = 4.13, *p* = 0.001), PLS-5 age equivalent EC scores (t(19) = 5.21, *p* < 0.001), PLS-5 age equivalent total scores (t(19) = 6.30, *p* < 0.001), PLS-5 growth scale value AC scores (t(19) = 4.10, *p* = 0.001), and PLS-5 growth scale value EC scores (z = 3.63, *p* < 0.001) from pre- to post-intervention. However, children with CIs did not demonstrate a significant change in standardized scores for any of the PLS-5 measures. Children with hearing aids also demonstrated a significant change in PLS-5 age equivalent AC scores (t(18) = 5.75, *p* < 0.001), PLS-5 age equivalent EC scores (t(18) = 5.52, *p* < 0.001), PLS-5 age equivalent total scores (t(18) = 6.96, *p* < 0.001), PLS-5 growth scale value AC scores (t(18) = 4.94, *p* < 0.001), and PLS-5 growth scale value EC scores (t(18) = 6.44, *p* < 0.001) from pre- to post-intervention. Furthermore, hearing aid children exhibited significant increases in PLS-5 standardized scores for EC (t(18) = 2.55, *p* = 0.020) and total language abilities (t(18) = 2.68, *p* = 0.015) following supplemental language therapy. There were no significant differences between children with hearing aids and CIs on changes in language scores pre- and post-supplemental language therapy.

Upon closer examination of hearing devices, children with hearing aids were found to show no significant differences between chronological age and all age equivalent PLS-5 scores following supplemental LSL therapy, as illustrated in Figure 4B. Meanwhile, children with CIs continued to demonstrate significantly lower age equivalent PLS-5 scores from their average chronological age (AC scores: (t(19) = 2.39, *p* = 0.027), EC scores: (t(19) = 2.34, *p* = 0.030), and total scores: (t(19) = 2.45, *p* = 0.024). Although children with hearing aids and CIs exhibited differences in how age equivalent PLS-5 scores compared to their chronological ages (as shown in Figure 4B), there were no significant differences in age equivalent PLS-5 scores for all language measures between these groups of children.

**Figure 5:**
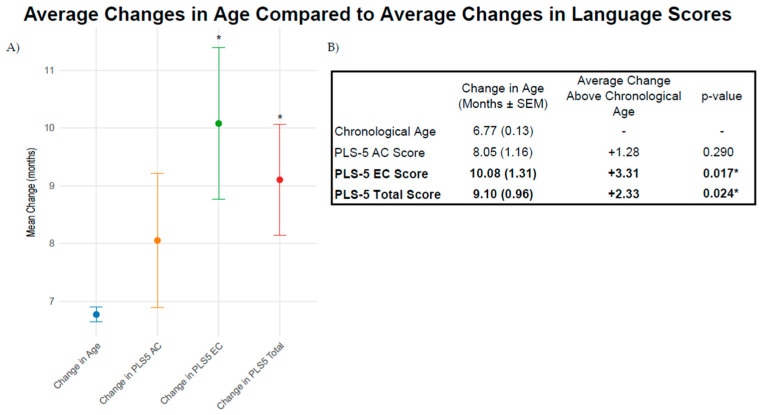
**Average changes in age compared to average changes in language scores.** (**A**). Mean (multicolored dots) change in chronological age (blue) compared to age equivalent language scores (AC: orange, EC: green, and Total: red). Error bars show the standard errors of the mean. (**B**). Table of average changes in chronological age and age equivalent language scores. Significantly greater improvements in expressive communication (EC) and total language scores than the change in chronological age were found over the duration of the study. * *p* < 0.05.

All children demonstrated greater gains in age equivalent PLS-5 EC scores (t(38) = 2.49, *p* = 0.017) and total scores (t(38) = 2.35, *p* = 0.024) throughout the duration of the study than would be expected for normal age developments. As shown in Figure 5, the change in PLS-5 age equivalent AC scores was not significantly greater than the children’s average change in chronological age. When examining children with CIs and hearing aids separately, changes in age equivalent scores were not significantly greater than changes in chronological age for each group.

### 3.3. Correlations

#### Language Measures and P1 Cortical Auditory Evoked Potentials

**Figure 6:**
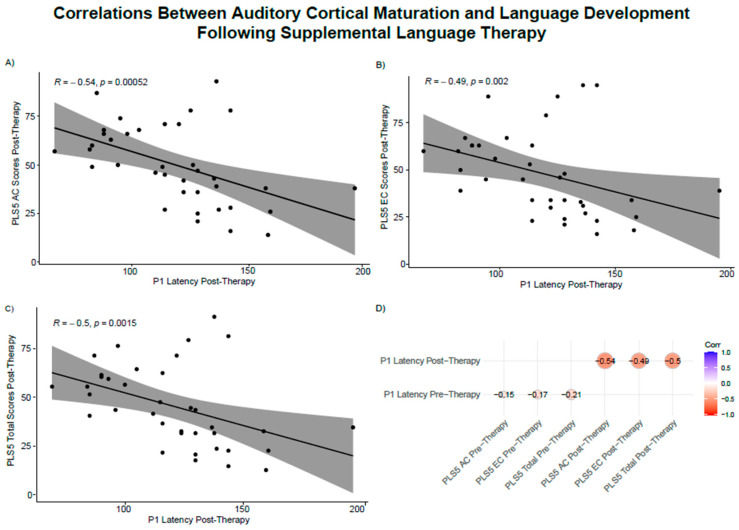
**P1 latencies are correlated with language scores following language therapy.** p1 responses were correlated with PLS-5 auditory comprehension scores (**A**), expressive communication scores (**B**), and total language scores (**C**) following supplemental language therapy. Longer P1 latencies, suggestive of a less mature auditory cortex, are associated with worse language abilities. (**D**) Correlogram of correlation coefficients between pre-language therapy P1 latencies and language outcomes and post-language therapy P1 latencies and language outcomes. The color and size of the circles indicate the strength and direction of the correlations. While there were no significant correlations prior to therapy, auditory cortex maturation as indexed by the P1 CAEP latency was negatively correlated with language development.

There were no significant correlations between P1 latencies and PLS-5 age equivalent scores prior to supplemental language therapy. However, following supplemental language therapy there were significant negative moderate correlations between P1 latencies and age equivalent PLS-5 AC scores (r = −0.54, *p* = 0.001), EC scores (r = −0.49, *p* = 0.002), and total scores (r = −0.50, *p* = 0.002). As shown in Figure 6, these correlations indicate that longer P1 latencies, reflective of less auditory cortical maturation, are associated with lower age equivalent language scores.

## 4. Discussion

Overall, this study found significant improvements in CAEP P1 responses with 66% of the children showing abnormal auditory cortical maturation (as indexed by P1 responses) prior to obtaining supplemental language therapy, while only 15% of children continued to have abnormal P1 responses at follow-up. Following supplemental LSL therapy, P1 latencies on average decreased greater than expected due to age-related maturation. Both CI children and children with hearing aids demonstrated significant decreases in P1 latencies in a subgroup analysis following supplemental therapy. A significant coinciding improvement in language outcomes was also noted along with these changes in P1 latencies. In approximately six months of therapy, the children in this study made 9 to 10 months of total language gains and expressive communication language gains. Interestingly, children with hearing aids may have derived greater benefits in language abilities from supplemental therapy than CI children as evidenced by a lack of significant difference between PLS-5 age equivalent scores and chronological age in only children with hearing aids at follow-up.

In a group of children with hearing aids and Cis, and below normal language development, following supplemental language therapy, all of the children showed improvements in auditory cortical maturation. These results are consistent with previously reported findings in which P1 latencies were found to significantly decrease following intervention in children with hearing aids and CIs [41]. In a large study of deaf children, Sharma et al. [3] established a sensitive period for auditory cortical maturation of 3.5 years. Within this period, the central auditory pathways are highly plastic and deaf children implanted before the age of 3.5 years were largely found to be able to overcome their initial lack of auditory stimulation to show cortical auditory development that is comparable to age matched normal hearing peers. However, children implanted beyond the age of 3.5 years were at increased risk for abnormal cortical auditory maturation regardless of the duration of CI use [2,3,42].

Interestingly, the children in the present study were enrolled many years after receiving treatment with hearing aids and CIs and language therapy, and despite their average age being beyond the sensitive period the results of this study suggest that neuroplastic changes still occur following supplemental language therapy later in childhood. In fact, for many of the children CAEP latencies following supplemental LSL therapy became age appropriate. In further support of neuroplasticity beyond the sensitive period, in both adult normal hearing listeners and CI users auditory training has resulted in changes of the CAEP response following short-term auditory training [43,44,45]. Changes in CAEP responses have also been noted in normal hearing adolescents undergoing listening therapy with a musical component [46]. Our findings extend reports of reductions in P1 latencies in CI children following auditory training [18], by demonstrating that the changes in P1 latencies in the children in this study following supplemental therapy were greater than the expected maturational changes for the children’s ages. Therefore, the results of this study along with previous studies indicate that CAEP measures can provide a useful objective measure of tracking aural rehabilitation outcomes.

As noted in previous studies involving children with hearing loss, prior to supplemental language therapy as a group, children in this study showed language skills significantly below that of their chronological age [21,24]. However, growth in language skills as measured by the PLS-5 following supplemental therapy also increased. The children in this study showed greater gains in total language scores and expressive communication than would be expected from normal development. These findings along with previous studies demonstrate the benefits of supplemental language therapy [22,23,47,48]. The children in this study received supplement therapy either in-person or via telehealth. Supplemental LSL therapy via telehealth has previously been shown to be noninferior to in-person services [37]. Therefore, with telehealth services it may be possible for more children to receive these benefits from supplemental language therapy. However, similar to the findings of Jackson and Schatschneider [23] the CI children in this study continued to show age equivalent language scores below that of their chronological age. Earlier hearing loss intervention has been associated with better speech and language outcomes [24,29,31,48,49,50], however, Tomblin et al. [24] noted that children fit with hearing aids later in childhood could still begin to catch up to their normal hearing peers as language development continued through six years of age further demonstrating that neuroplasticity continues throughout the lifespan.

Children with hearing aids and CIs demonstrated differing patterns of language development and cortical maturation. Only children with CIs exhibited all language measures below their chronological age at pre- and post-supplemental language therapy. This pattern of language development corresponded with children with CIs showing more abnormal P1 responses for their chronological age at baseline, although this finding was not significant. This was despite the fact that in terms of audibility, children with hearing aids showed significantly poorer aided speech testing (38dB HL) than the children with CIs (27dB HL). Despite the differences in aided testing, children with CIs have a more severe to profound degree of hearing loss, compared to children with hearing aids, whose hearing loss typically is in the mild to moderate range. Previous studies have reported language abilities are negatively impacted by greater degrees of hearing loss, pre-amplification language abilities, and the presence of multiple disabilities [24,49,51].

Interestingly, 55% of children in the CI group and 36.84% of children in the HA group had additional disabilities. Importantly, previous studies have shown that children with worse pre-treatment language abilities and greater degrees of hearing loss have been noted to obtain the greatest benefits in language abilities post-treatment [51]. It is also important to note that the aforementioned intervention strategies, focusing more heavily on oral-aural rehabilitation and spoken language outcomes, are not the only rehabilitation methods available. There are other rehabilitation strategies, such as sign language which is popular within the Deaf community. Sign language in deaf CI users has been demonstrated to have positive benefits on spoken language abilities [52,53]. In fact, Delcenserie et al. reported that more sign language use pre-and post-cochlear implantation was associated with better spoken language outcomes compared to CI children who did not use any sign language [52]. It may be that exposure to sign language mitigates some of the effects of early language deprivation in deaf children and can act as a scaffold for spoken language development [52,53]. While possible mediation effects of sign language on auditory cortical maturation and language development were beyond the scope of this study, future studies may wish to examine how the amount of sign language exposure may affect these results.

Interestingly, only children with hearing aids showed significant improvement in standardized scores, while both children with hearing aids and CIs demonstrated significant increases in growth scale value scores. Daub et al. [51] noted a very similar pattern of no significant change in standardized scores, but changes in growth scale value scores in a sample of young children pre- and post-hearing aid fitting. The authors concluded that their results suggested personal language growth that is however not sufficient enough to change the children’s language standings relative to their normal hearing peers. Therefore, it could be that while children with hearing aids in this study began to catch up to their normal hearing peers following supplemental therapy, the children with cochlear implants made improvements, but not at a fast enough rate to change their language standings compared to normative data. It is possible that had we followed up with these children over a longer duration, the children with CIs may have also demonstrated growth in standardized scores. Future longitudinal studies with larger sample sizes are warranted to fully explore language developmental trajectories as a function of hearing device following supplemental language therapy.

In terms of cortical reorganization, Kral and Sharma [54] have also described a dynamic system in which cortical reorganization driven by changes in the balance of inhibitory and excitatory inputs titrated by the degree of hearing loss severity. Specifically, auditory input to the system is greater with milder degrees of hearing loss resulting in more typical cortical processing and less reorganization. However, as found in previous studies, there were no significant differences when directly examining between device group cortical maturation outcomes and language outcomes in our study [25,26,27]. Yet, when compared to normative values only the children with CIs continued to show language abilities below their chronological age at follow-up. The present findings may suggest that comparing to age normative values and chronological age may reflect nuances in language differences in development between children with CIs and children with hearing aids.

P1 latencies post-supplemental language therapy were negatively correlated with PLS-5 total language scores, AC scores, and EC scores. This suggests that more mature cortical auditory maturation is related to better language abilities which aligns with previous research on P1 latency development and language development in children with CIs [7,8,9,19,41]. As we demonstrate in this study, a potential objective biomarker that predicts language skills and changes with language therapy could be important in guiding clinical decision making. Finally, if future randomized clinical trials demonstrate the benefits of supplemental language therapy, then technological innovations such as telemedicine or digital therapists/robots may be leveraged for service delivery.

## 5. Limitations of the Study

Limitations of this study include the retrospective design and the small sample size, in particular for subgroup analyses comparing differences in outcomes as a function of hearing device. Future studies should aim to confirm the findings reported here with larger sample sizes. While the results of this study show promise, additional studies are needed to determine if the effects reported are sustained over time through the use of a longitudinal study design rather than only examining pre- and post-intervention outcomes. This study included a specific population of children, specifically English-speaking children with hearing loss displaying difficulties with language development. Future research should examine the generalizability of these results for children from more diverse backgrounds including, but not limited to those not self-identifying as displaying language difficulties. No control group was included in this study due to its retrospective nature, however, it would be challenging to withhold therapy services from children with hearing loss to obtain a proper control group and the measures collected enabled comparisons to age-matched normative values or chronological age in order to minimize the impacts of this limitation. Furthermore, a comparison to normative data is commonly utilized in the hearing loss population for both outcomes of cortical auditory maturation and language development [5,6,8,10,11,12,13,14,15,21,22,23,24,51]. This exploratory study utilized only one type of supplemental language therapy, specifically listening and spoken language (LSL) therapy. Therefore, it is difficult to determine if the effects reported from this study are driven by the type of therapy or the amount of supplemental therapy. This study found both language gains, as well as auditory cortical maturation gains with the inclusion of children with self-reported multiple disabilities with supplemental therapy. However, future studies may benefit from systematically exploring the impacts of multiple disabilities on speech-language therapy outcomes. While the type of supplementary therapy was held constant for all of the children, we were not able to hold constant the type of original therapy the children were receiving prior to and during enrollment in the study. Thus, future studies are warranted to examine the effects and potential interactions of different types of therapy and therapy dosages across hearing treatment devices.

## 6. Conclusions

The P1 CAEP response is an objective biomarker of auditory cortical development. The present findings demonstrate that the P1 response is not only sensitive to hearing loss intervention with hearing aids and CIs, but also shows changes in response to supplemental language therapy for children with hearing loss. Improvements in cortical maturation were co-incident with increases in language skills. Supplemental language therapy resulted in more typical auditory cortical maturation and language abilities, which were correlated with each other. In a preliminary subgroup analysis, children with hearing aids showed better language outcomes after receiving supplemental therapy than children with CIs. Overall, the results from this study suggest that the brain maintains its capacity for neuroplastic changes well into childhood. The P1 CAEP biomarker may provide a useful way to measure neuroplastic changes in children with hearing loss as a function of hearing treatments and/or language therapy.

## Figures and Tables

**Table 1:** Participant demographic information.

		All Children	Hearing Aid Users	CI Users
Number of Children		39	19	20
Demographics	Age (mos; mean ± SEM)	48.26 ± 2.88	45.47 ± 2.97	50.90 ± 2.79
	Gender (% of females)	51.28	47.37	55.00
	Maternal Education (% with college degree)	61.54	47.37	75.00
	Multiple Disabilities (%)	46.15	36.84	55.00
	Sign Language Use (%)	81.97	78.95	85.00
Hearing Rehabilitation	Age of First Device Use (mos; mean ± SEM) ^1^	14.56 ± 2.81	19.05 ± 3.14	10.30 ± 2.32
	Age of First Cochlear Implant (mos; mean ± SEM)	-	-	24.25 ± 3.73
	Hours of Device Use at Baseline (mean ± SEM)	12.05 ± 0.71	10.79 ± 0.65	13.25 ± 0.72
	Hours of Device Use at Follow-Up (mean ± SEM)	11.82 ± 0.33	11.42 ± 0.35	12.20 ± 0.31
	Aided Testing (dB HL)	32.01 ± 2.04	37.67 ± 2.44	26.64 ± 1.04 *
Listening and Spoken Language Therapy	In person (%)	56.41	42.11	70.00
	Telehealth (%)	43.59	57.89	30.00
	Length (mos; mean ± SEM)	5.79 ± 0.08	5.75 ± 0.51	5.83 ± 0.54

^1^ age of first hearing device (i.e., hearing aid or cochlear implant) * *p* < 0.05.

## Data Availability

The data used in this study are available from the corresponding author upon reasonable request. The data are not publicly available due to privacy concerns.
